# Hydroxycitric Acid Alleviated Lung Ischemia-Reperfusion Injury by Inhibiting Oxidative Stress and Ferroptosis through the Hif-1α Pathway

**DOI:** 10.3390/cimb45120616

**Published:** 2023-12-08

**Authors:** Zi-Long Lu, Cong-Kuan Song, Shi-Shi Zou, Shi-Ze Pan, Kai Lai, Ning Li, Qing Geng

**Affiliations:** Department of Thoracic Surgery, Renmin Hospital of Wuhan University, Wuhan 430000, China

**Keywords:** hydroxycitric acid, lung ischemia-reperfusion injury, ferroptosis, HIF-1α

## Abstract

Lung ischemia-reperfusion injury (LIRI) is a prevalent occurrence in various pulmonary diseases and surgical procedures, including lung resections and transplantation. LIRI can result in systemic hypoxemia and multi-organ failure. Hydroxycitric acid (HCA), the primary acid present in the peel of Garcinia cambogia, exhibits anti-inflammatory, antioxidant, and anticancer properties. However, the effects of HCA on LIRI remain unknown. To investigate the impact of HCA on LIRI in mice, the mice were randomly divided into four groups: the control group, the I/R model group, and the I/R + low- or high-dose HCA groups. Human umbilical vein endothelial cells (HUVECs) were subjected to hypoxia for 12 h followed by reoxygenation for 6 h to simulate in vitro LIRI. The results demonstrated that administration of HCA effectively attenuated lung injury, inflammation, and edema induced by ischemia reperfusion. Moreover, HCA treatment significantly reduced malondialdehyde (MDA) and reactive oxygen species (ROS) levels while decreasing iron content and increasing superoxide dismutase (SOD) levels after ischemia-reperfusion insult. Mechanistically, HCA administration significantly inhibited Hif-1α and HO-1 upregulation both in vivo and in vitro. We found that HCA could also alleviate endothelial barrier damage in H/R-induced HUVECs in a concentration-dependent manner. In addition, overexpression of Hif-1α counteracted HCA-mediated inhibition of H/R-induced endothelial cell ferroptosis. In summary, these results indicate that HCA alleviated LIRI by inhibiting oxidative stress and ferroptosis through the Hif-1α pathway.

## 1. Introduction

Lung ischemia-reperfusion injury (LIRI) is widely recognized as the primary cause of primary graft failure (PGD), which represents the most prevalent factor leading to early mortality following lung transplantation and chronic lung graft dysfunction [1]. The pathophysiological process of LIRI is characterized by the involvement of oxidative stress, infiltration of inflammatory cells, damage to lung epithelial and endothelial cells, and impairment of blood–gas barrier function [2]. Despite significant advancements in understanding LIRI, its precise mechanism remains incompletely elucidated and lacks targeted therapeutic interventions [3].

Iron is an essential trace metal ion for the human body and plays a pivotal role in numerous physiological functions. Ferroptosis, characterized by the accumulation of lipid peroxide and intracellular ROS through the Fenton reaction [4], represents an iron-dependent form of cell death. Various pathways can trigger ferroptosis-inducing factors to act on glutathione peroxidase, leading to the buildup of lipid ROS within cells and subsequently inducing oxidative cell death [5]. Morphologically, mitochondrial damage, including condensation of the mitochondrial membrane and the reduction or disappearance of cristae, can be observed [6]. Recent studies have unveiled that ferroptosis is intricately associated with pathophysiological processes involved in a range of diseases, including tumors, nervous system disorders, cardiovascular disease, kidney injury, and ischemia-reperfusion injury (IRI) [7]. Previous investigations have demonstrated that during the reperfusion period after I/R, ferroptosis occurs due to severe impairment in endogenous ROS scavenging mechanisms that fail to effectively eliminate ROS-mediated I/R injury [8].

HCA, a derivative of citric acid, is the principal acid present in Garcinia cambogia. Garcinia cambogia, native to Southeast Asia, has a peel that is used as a food preservative, flavoring agent, or bulking agent [9]. Currently, it is predominantly employed as a weight-loss supplement. Research studies have demonstrated that HCA exhibits anti-obesity properties by enhancing fat oxidation and reducing fat production [10]. Acting as a potent inhibitor of adenosine triphosphate citrate lyase, HCA effectively hinders the conversion of citric acid to acetyl coenzyme A [11]. An increasing body of evidence suggests that HCA possesses broad pharmacological activities, including inhibition of fatty acid synthesis, anti-tumor effects, and amelioration of inflammation and oxidative stress [12,13]. In this study conducted on a glyoxylate-induced mouse model, it was observed that HCA prevented renal damage and calcium oxalate crystal deposition [14]. Furthermore, studies have indicated that HCA can inhibit choroidal neovascularization through suppression of hypoxia-inducible factor-1 alpha (HIF-1α) in the treatment for age-related macular degeneration [15].

Based on these investigations, we postulated that HCA possesses the potential to mitigate LIRI by inhibiting oxidative stress and reducing ROS production. Consequently, the objective of this study is to experimentally validate this hypothesis both in vitro and in vivo.

## 2. Materials and Methods

### 2.1. Reagents and Antibodies

Hydroxycitric acid (HCA, HY-16007, HPLC: 98.11%), ferrostatin-1 (Fer-1, HY-100579) and erastin (HY-15763) were purchased from MCE (Shanghai, China). Antibodies against HIF-1α (#36169, 1:1000), HO-1 (#26416, 1:1000), ZO-1 (#13663, 1:200), and VE-cadherin (#2500, 1:400) were purchased from Cell Signaling Technology, Inc.^®^(Danvers, MA, USA), and GPX4 (ab125066, 1:2000), SLC7A11 (ab175186, 1:2000), ACSL4 (ab155282, 1:10,000), and FTH1 (ab75972, 1:1000) were purchased from Abcam (Cambridge, MA, USA). Antibodies against β-actin (CL594-66009, 1:1000) and horseradish peroxidase-conjugated goat anti-rabbit (SA00001-2, 1:1000) or anti-mouse secondary antibodies (SA00001-1, 1:1000) were purchased from Proteintech Group (Wuhan, China). Malondialdehyde (MDA) assay kit (A003-1-2), Superoxide Dismutase (SOD) assay kit (A001-3-2), Total glutathione/Oxidized glutathione assay kit (A061-1-1), and tissue iron assay kit (A039-2-1) were purchased from Jiancheng Bioengineering Institute (Nanjing, China). The ROS Fluorescent Probe Kit was used to detect tissue (KeyGEN, Nanjing, China) and cell (Biosharp, Hefei, China) ROS.

### 2.2. Animal Treatments

All animal procedures were conformed to the Guide for the Care and Use of Laboratory Animals. Male wild-type C57BL/6 mice (6–8 weeks old) were obtained from Hubei Province Experimental Animal Center (Wuhan, China, ethic code: 20220902B). All mice were weighed, coded, and randomly assigned to each group; the number of mice in each group was five. The mice were divided into four groups including the sham group, I/R model group, I/R + low-dose HCA (200 mg/kg) group, and I/R + high-dose HCA (400 mg/kg) group. Mice were anesthetized by peritoneal injection of 3% sodium pentobarbital (50 mg/kg). The mice were ventilated using a rodent ventilator (MiniVent Harvard Apparatus, Holliston, MA, USA) with the ventilator frequency set to 130–150 breaths/min, the tidal volume set to 150–200 mL/min, and the inspiratory/expiratory ratio set to 1:1.5. During the operation, the mice were placed on a thermostatic pad to ensure constant body temperature. A non-invasive clamp was used to occlude the left pulmonary hilum to induce lung ischemia, and mice in the sham group underwent left thoracotomy without occlusion of the pulmonary hilum with mechanical ventilation maintained for 3 h. After endotracheal intubation, mice in the I/R group were subjected to left thoracotomy followed by occlusion of the left pulmonary hilum with a non-invasive vascular clamp for 30 min and reperfusion of the pulmonary hilum, followed by ventilation for 2 h as described [16]. Mice in the I/R + HCA group received different concentrations of HCA (200 mg/kg/d, 400 mg/kg/d) by gavage one week before surgery, and the other operations were performed identically to those in the I/R group. These mice were sacrificed after 2 h of reperfusion, and lung tissues were collected for pathological and biochemical experiments. The present study was approved by the Animal Use Committees of Renmin Hospital, Wuhan University.

### 2.3. Hematoxylin–Eosin (H&E) Staining

The tissues were fixed in 4% paraformaldehyde, embedded in paraffin, and cut into 5 μM thick sections, followed by staining with hematoxylin–eosin stain.

### 2.4. Lung Injury Score

The severity of lung damage was assessed independently by two technicians using a semiquantitative scoring standard. The scoring system consisted of four components: pulmonary interstitial edema, neutrophil infiltration, alveolar edema, and alveolar congestion. Alveolar septal thickening, hemorrhage, and edema were characterized as follows: absent (score = 0), mild (score = 1), moderate (score = 2), severe (score = 3), and very severe (score = 4).

### 2.5. Lung Wet/Dry Mass Ratio

Half of the left lung tissue was removed and weighed immediately after reperfusion to determine wet weight. The lung tissues were then placed in a 60 °C oven for 96 h and reweighed until a constant mass was reached. The wet/dry mass ratio was calculated to assess the severity of pulmonary oedema.

### 2.6. Elisa

Interleukin-1β (IL-1β), interleukin-6 (IL-6), tumor necrosis factor-α (TNF-α), malonaldehyde (MDA), glutathione (GSH), and superoxide dismutase (SOD) levels, all obtained from Nanjing Jiancheng Bioengineering Institute, were detected by the corresponding kits in accordance with the manufacturer’s protocols. The tissue iron concentrations were also determined as instructed.

### 2.7. Iron Content Assay

Lung tissues were homogenized in saline and PBS followed by centrifugation. Protein concentration was measured using the BCA protein assay kit. Blood samples were collected, allowed to stand for 1 h, and centrifuged; then, the serum was collected for assay. Iron concentrations in blank (ddH_2_O), iron standard solution, and test samples (supernatant and serum) were determined using the Iron Assay Kit (TC1015, Leagene, Beijing, China) according to the manufacturer’s instructions. The reaction mixture was incubated at room temperature for 15 min. The absorbance at 562 nm was measured using a microplate reader.

### 2.8. Cell Culture and Cell Treatment

HUVECs were obtained from the American Type Culture Collection (ATCC, Manassas, VA, USA) and were cultured in Dulbecco’s modified Eagle’s medium (DMEM) (Gibco, Carlsbad, CA, USA) supplemented with 10% fetal bovine serum (FBS) (Gibco, California, USA) and 1% penicillin/streptomycin in an atmosphere of 5% CO_2_ humidified incubator at 37 °C. When the density of the cells reached 90%, we trypsinized the cells with 0.05% trypsin/1 mM EDTA (HyClone, Logan, UT, USA) and plated them onto 6-well culture plates (105 cells per well) for experimental treatments. The cells were randomly divided into six groups: (1) the sham group; (2) H/R model group; (3) H/R + low-dose HCA (100 μM) group; (4) H/R + high-dose HCA (200 μM) group; (5) erastin 4 μM group [17]; and (6) H/R + Fer-1 1 μM group [18]. We used these inhibitors or the vehicle DMSO at nontoxic concentrations that had no effect on morphology or cell viability of HUVEC cells.

We also developed H/R HUVECs models: When cell confluency reached approximately 85%, normal cell culture medium was renewed with serum-free and glucose-free DMEM. The cells were placed in a tri-gas incubator containing 95% N_2_ and 5% CO_2_ for 12 h under hypoxic conditions. Then, the culture medium was replaced with DMEM (Gibco, USA) containing glucose, 1% penicillin–streptomycin, 10% FBS, and 4 mM L-glutamine for 6 h to construct the H/R cell model [19]. The cells were collected for subsequent experiments. Erastin and Fer-1 were dissolved into DMSO with different concentrations. HCA was dissolved into PBS with different concentrations. Then, they were used to treat HUVECs in vitro for 24 h before H/R stimulation in experiments.

### 2.9. Assay of Cell Viability

The HUVEC cells were plated on 96-well plates for 12 h and then cultured with HCA at the corresponding dose for 24 h. The CCK8 assay kit (#ab228554, Abcam) was used to detect cell viability. HUVECs were cultured in 96-well plates; after appropriate treatment, 10 mL of the CCK-8 reagent was mixed and inoculated at 37 °C for 2 h; then, the normal, H/R and HCA pre-treated HUVECs were washed twice with PBS; then, cell viability was detected at a wavelength of 450 nm using a SpectraMax (Molecular Devices, Sunnyvale, CA, USA) absorbance reader microplate.

### 2.10. Wound Healing Assays

HUVEC cells were seeded in 6-well plates (2 × 10^5^ cells/well) and cultured to 85% confluence. The cell monolayer was wounded by manual scraping with 200 μL pipette tip. Cells were washed with phosphate-buffered saline (PBS). Cells were incubated in DMEM supplemented with 1% FBS. Cell migration was observed using an Olympus BX51 microscope (magnification, ×100; Olympus BX51; Olympus, Tokyo, Japan) at four preselected time points (0, 6 h, 12, and 24 h). ImageJ software (version 1.53c, National Institutes of Health, Bethesda, MD, USA) was used to quantitatively calculate the area covered by the cells.

### 2.11. Transwell Assay

HUVEC cells were inoculated in the upper chamber of a Transwell (8 μM wells, Corning, Somerville, MA, USA) with serum-free ECM. The lower chamber was filled with complete medium containing 10% serum. After waiting for the cells to migrate freely for 24 h, the cells were fixed in 4% PFA, washed twice with PBS, and subsequently incubated with crystal violet staining solution (Solarbio, Beijing, China) for 10 min. For each sample, at least three random fields of view were selected and imaged using a light microscope (Leica DM3000, Wetzlar, Germany). The number of migrated cells was counted using ImageJ software (version 1.53c, NIH, Bethesda, MD, USA).

### 2.12. ROS Assay

The medium was replaced with PBS, and the cells were incubated with 500 μL DCFH-DA probe solution (10 μM, Beyotime Biotech, Shanghai, China) at 37 °C for 20 min after H/R model induction and drug treatment. Subsequently, the cells were washed once in serum-free DMEM and maintained in 500 μL medium. A fluorescence microscope (Olympus U-RGLT50, Tokyo, Japan) was used to determine ROS production. The mean fluorescence intensity was analyzed using ImageJ software.

### 2.13. Transmission Electron Microscopy (TEM)

After induction of the H/R model and appropriate drug treatment, samples of HUVEC cells were collected and fixed in glutaraldehyde. The fixed cells were then dehydrated through a graded ethanol series and sectioned into ultrathin slices. Sections were then stained with uranyl acetate and lead citrate and examined with a HT-7500 transmission electron microscope (Hitachi Co., Ltd., Tokyo, Japan).

### 2.14. Western Blot and Real-Time Fluorescence Quantitative PCR

The lung tissues and treated HUVEC cells were lysed using a RIPA buffer. The protein concentrations were detected by the BCA Protein Assay Kit. Protein electrophoresis was performed in SDS/PAGE gels and transferred to membranes. The primary antibody was incubated overnight at 4 °C in the refrigerator after 2 h of closure, and the secondary antibody anti-rabbit-HRP (1:3000; Proteintech) or anti-mouse-HRP (1:3000; Proteintech) was incubated at room temperature for 2 h the next day. The protein bands were visualized with the enhanced chemiluminescence Western blotting detection system (Bio-Rad, Santa Rosa, CA, USA).

Real-time quantitative PCR to detect the expression of mRNA of related molecules: The TRIzol method was used to extract total mRNA from tissues or cells, and 2 μg of the obtained mRNA was reverse-transcribed to synthesize cDNA. Quantitative real-time PCR was performed using EnTurbo™ SYBR Green PCR SuperMix (ELK Biotechnology, EP001, Wuhan, China). The expression levels of target genes were uniformly normalized to Actin. All primers used in this study are listed in Supplementary Table S1.

### 2.15. Flow Cytometry

The cell death rate was detected using the annexin V–fluorescein isothiocyanate (FITC)/propidium iodide (PI) apoptosis detection kit (C1062M, Beyotime Biotechnology, Shanghai, China). A total of 1 × 10^6^ cells/mL were washed twice with cold PBS. The cells were then gently resuspended in 195 μL annexin V–FITC binding solution. The cells were then incubated with 5 μL of annexin V–FITC and 10 μL of PI for 20 min at a temperature of 37 °C in the dark. The cell death rate was quantified using a flow cytometer (FACSVerse/Calibur/AriaIISORP, BD Biosciences, San Jose, CA, USA).

### 2.16. Mitochondrial Membrane Potential (MMP) Assay

HUVEC cells were plated on 6-well plates. A JC-1 fluorescent probe (CAS 3520-43-2, Abcam) was used to measure MMP in HUVEC cells. Cells were incubated with 5 μM JC-1 for 20 min in the dark at 37 °C. After the cells were washed with JC-1 staining buffer (1X), the cells were incubated in DMEM supplemented with 1% FBS, and then, the cell images were detected using a fluorescence microscope (TE-2000, Nikon Co., Ltd., Tokyo, Japan).

### 2.17. Mitochondrial Ferrous Iron (Fe^2+^) Determination

The Mito-Tracker Green fluorescent probe (Beyotime, Shanghai, China) was used to examine the mitochondrial Fe^2+^of HUVEC cells. After the medium was removed, the cells were treated with 5 μM Mito-FerroGreen working solution for 30 min in a dark room. Then, we removed the working fluid and added fresh DMEM medium to the cells. Finally, the cell images were examined using a confocal microscope (TCS-SP2, Leica, Germany) at Ex and Em wavelengths of 488 nm and 510–550 nm, respectively.

### 2.18. Immunofluorescence Staining

After various stimuli, HUVEC cells were fixed with 4% paraformaldehyde for 30 min, rinsed three times with PBS, and then treated with 0.2% Triton X-100 (Solarbio, Beijing, China) for 20 min at room temperature. Cells were blocked in 5% BSA (Servicebio, Wuhan, China) for 30 min at room temperature after three washes with PBS. Cells were then incubated with primary antibodies against vascular endothelial cadherin (VE-cadherin) (1:400) and zona occludens-1 (ZO-1) (1:200) overnight at 4 °C. The samples were then incubated with Cy3-conjugated anti-rabbit secondary antibody (1:200, Servicebio, China) at 37 °C for 1 h, followed by DAPI staining for 5 min, followed by four washes with PBS. Images were captured with a fluorescence microscope (Olympus IX51, Tokyo, Japan), and analysis was performed with ImageJ software.

### 2.19. Cell Transfection

Cell transfection was performed when cells were at 70–80% confluence. HUVEC cells were infected with lentiviruses (Genechem, Shanghai, China) expressing short hairpin (sh) RNA (1 × 10^9^ TU/mL, MOI of 10:1) to knock down HIF-1α (5′-CTGATAACGTGAACAAATA-3′) expression in the presence of polybrene (2 μg/mL) according to the manufacturer’s instructions. Lent-OE-HIF-1α (HanBio, Shanghai, China) was transfected into HUVECs using Lipofectamine 2000 (Invitrogen, Waltham, MA, USA) according to the manufacturer’s protocols to establish in vitro HIF-1α-expression models. After infection, the levels of HIF-1α were determined by Western blotting, and the data were used for the construction of the H/R model.

### 2.20. Molecular Docking

The two-dimensional structure of HCA was explored by the PubChem database (https://pubchem.ncbi.nlm.nih.gov/, Visited on 10 September 2023), and its three-dimensional model was produced by ChemOffice software(version 2012). HIF-1α (PDB ID: 8HE3) was downloaded from the RCSB Protein Data Bank (https://www.rcsb.org/, Visited on 10 September 2023) and imported into PyMOL software (version 3.11.2), in which these molecules were treated by the procedures of removing water and adding hydrogen. At last, the results of molecular docking were evaluated based on -CDOCKER energy scores [20].

### 2.21. Statistical Analysis

All analyses were performed in SPSS 22.0 and GraphPad Prism 8 software. Data are expressed as mean  ±  standard deviation (SD). One-way ANOVA analyzed differences among multiple groups. *p* < 0.05 was considered statistically significant.

## 3. Results

### 3.1. HCA Pretreatment Attenuates I/R-Induced Lung Injury and Inflammation in Mice

**Figure 1:**
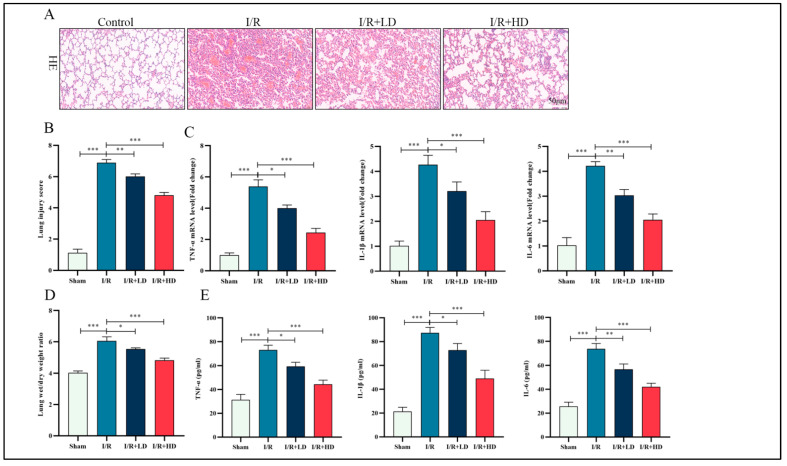
HCA protects against I/R-induced pathological injury of murine lung tissue. (**A**) H&E staining of murine lung tissues (bar: 50 μM). (**B**) Semiquantitative histological scores of lung injury in groups described in panel. (**C**) The mRNA level of TNF-α, IL-1β, and IL-6 in the indicated groups. (**D**) Lung wet/dry ratio in the indicated groups. (**E**) The levels of TNF-α, IL-1β, and IL-18 in the lung tissues determined by ELISA (*n* = 5, * *p* < 0.05; ** *p* < 0.01; *** *p* < 0.001).

The molecular structure of HCA is depicted in Figure S1A. We chose two doses of HCA, namely 200 mg/kg and 400 mg/kg, and administered them by gavage; no cardiac, hepatic, or renal toxicity was observed in mice, even at the higher doses (Figure S1B–E). Post-surgical evaluation using H&E staining revealed significant pathological injury caused by I/R, including interstitial edema, alveolar hemorrhage, and extensive infiltration of inflammatory cells when compared to the control group. However, pretreatment with HCA effectively alleviated the I/R-induced pathological injury in murine lung tissue (Figure 1A,B), particularly at the high dose, which exhibited optimal efficacy. Furthermore, HCA pretreatment also reduced pulmonary edema induced by I/R, as evidenced by a lower wet/dry ratio of the lungs (Figure 1D). Excessive activation of the inflammatory response is a crucial pathological feature in LIRI [21]. The expression of inflammatory cytokines TNF-α, IL-1β, and IL-6 in lung tissue was assessed using ELISA. Additionally, mRNA levels of pro-inflammatory cytokines including TNF-α, IL-1β, and IL-6 were measured. As depicted in Figure 1C,E, there was a significant inhibition of proinflammatory cytokine expression in the I/R + HCA group compared to the I/R group, with a more pronounced relief observed in the high-dose HCA group. Henceforth, these findings suggest that HCA mitigates LIRI and attenuates the inflammatory response in mice.

### 3.2. HCA Demonstrated Inhibitory Effects on Ferroptosis in Mice with LIRI

**Figure 2:**
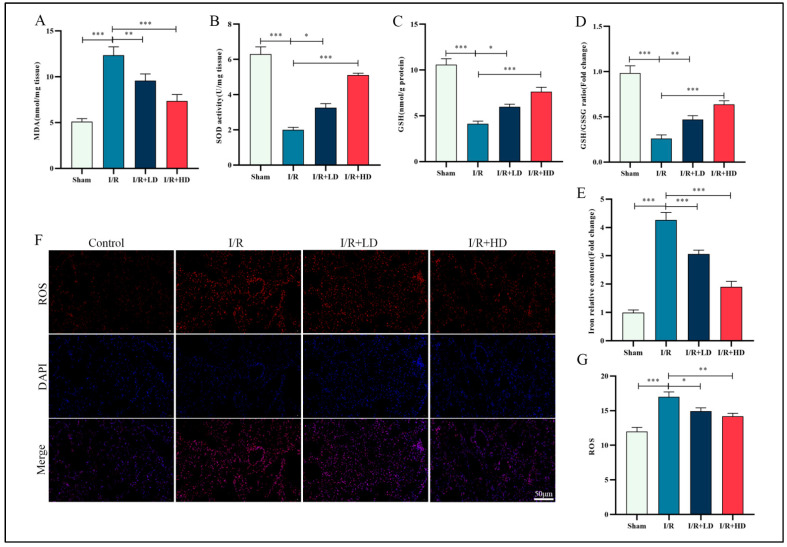
HCA inhibits oxidative stress in murine lung during LIRI. (**A**–**E**) Relative MDA, SOD, GSH, and GSH/GSSG ratio and iron levels of murine lung tissue. (**F**,**G**) Representative images of fluorescence probe for ROS and its statistical results (*n*  =  5) in lung tissue (bar: 50 μM).* *p* < 0.05; ** *p* < 0.01; *** *p* < 0.001.

Oxidative stress plays a pivotal role in lung injury, particularly during reperfusion. Ferroptosis, characterized by iron accumulation and lipid peroxidation, represents an oxidative-stress-dependent regulated cell death pathway [22]. The expression levels of oxidative-stress-related markers including MDA, SOD, GSH, and ROS were assessed using corresponding assay kits. We observed that the expressions of MDA were elevated in the I/R group compared to the control group; conversely, the expressions of SOD, GSH, and GSH/GSSG ratio were significantly reduced. Notably, administration of low or high doses of HCA resulted in decreased MDA levels and increased expressions of SOD, GSH, and GSH/GSSG in the I/R group (Figure 2A–D). Furthermore, HCA effectively inhibited iron content in the I/R group (Figure 2E). Immunofluorescence staining revealed that HCA prominently attenuated I/R-induced ROS elevation (Figure 2F,G). Collectively, the experimental findings suggest that HCA mitigates I/R-induced oxidative stress and iron accumulation.

### 3.3. The Inhibitory Effect of HCA on Ferroptosis in LIRI Was Mediated through the Suppression of Hif-1α and HO-1

**Figure 3:**
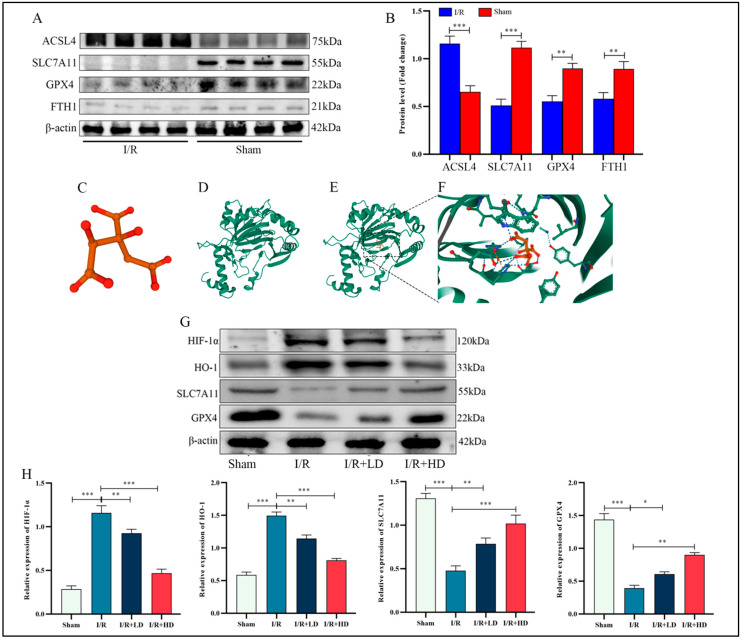
HCA inhibits ferroptosis in murine lung during LIRI. (**A**) Western blot showing the protein expression related to ferroptosis. (**C**) The three-dimensional model of HCA. (**D**) The three-dimensional model of Hif-1α. (**E**,**F**) The -CDOCKER energy between HCA and its potential targets. (**G**) Western blot showing the protein expression of HIF-1α, HO-1, SLC7A11, and GPX4. (**B**,**H**) Quantification of the results of (**A**,**G**). * *p* < 0.05; ** *p* < 0.01; *** *p* < 0.001.

It is widely acknowledged that the glutathione (GSH)-dependent antioxidant enzyme GPX4 plays a pivotal role in impeding ferroptosis. Subsequently, Western blot analysis was conducted to detect the expression of ferroptosis-related proteins. Our findings revealed that compared with the control group, ACSL4 expression was significantly elevated, while SLC7A11, GPX4, and FTH1 expressions were markedly reduced in the I/R group (Figure 3A,B). Previous studies have demonstrated therapeutic effects on age-related macular degeneration by inhibiting Hif-1α [23]. The three-dimensional structure of HCA is depicted in Figure 3C. To assess candidate drug affinity for its target, we performed a molecular docking analysis, which showed visible hydrogen bonds and strong electrostatic interactions between HCA and its protein target Hif-1α. For HCA, binding energy to Hif-1α was −4.378 kcal/mol, indicating highly stable binding (Figure 3C–F). In a dose-dependent manner after administration of HCA in the I/R group, SLC7A11 and GPX4 expression increased, while both Hif-1α and its downstream target gene HO-1 decreased (Figure 3G,H). We tentatively conclude that inhibition of ferroptosis occurs through administration of HCA during LIRI by suppressing both Hif-1α and HO-1.

### 3.4. HCA Enhanced HUVEC Migration Capacity and Ameliorated H/R-Induced Impairment of Endothelial Barrier Function

**Figure 4:**
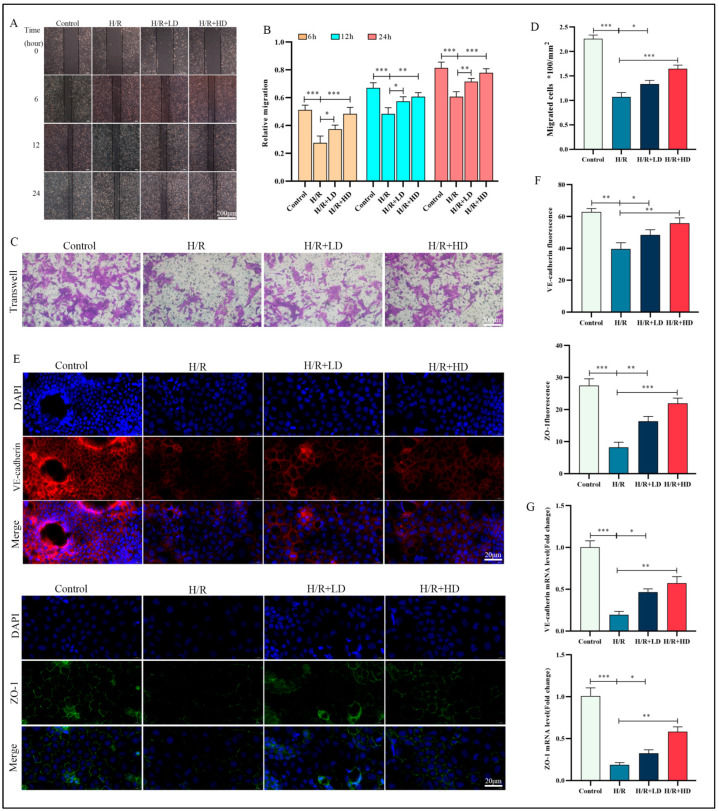
HCA promoted the migration of HUVEC and alleviated H/R-induced damage of endothelial barrier function. (**A**) The scratch assays were performed to detect the migration ability of HUVEC cells treated with HCA at different concentrations (100 and 200 μM). (**B**) Quantification of the results of (**A**). Results represent at least three independent experiments. (**C**,**D**) Representative images of the Transwell assay and the quantified number of migrated cells. Scale bar = 200 μM. (**E**) Expression of VE-cadherin and ZO-1 in HUVEC after HCA treatment was analyzed by immunofluorescent staining. Scale bar is 20 μM. Red or green, cells labeled with VE-cadherin and ZO-1; blue, DAPI. All data were compared with control group and presented as mean ± SEM (*n* = 5 in each group). (**F**) Quantification of the results of (**E**). (**G**) The mRNA level of VE-cadherin and ZO-1 in the indicated groups.* *p* < 0.05; ** *p* < 0.01; *** *p* < 0.001.

The vascular endothelium consists of a continuous monolayer of endothelial cells and plays crucial roles in maintaining pulmonary circulation. It is the primary target of I/R injury, which can lead to severe pulmonary dysfunction [24]. Based on our in vivo findings, we investigated whether HCA could prevent H/R-induced cell damage in HUVECs. We employed various concentrations of HCA for pretreatment, as described in a previous study [25], and assessed cell viability using the CCK-8 test kit. Our results showed that HCA did not induce significant cell death at concentrations up to 200 μM; however, slight toxicity was observed at 250 μM (Figure S2A). The results also indicated that H/R could decrease cell viability, with the most pronounced effect observed at 6 h of reoxygenation. However, this detrimental effect was rescued by pretreating HUCEC cells in vitro with a concentration of 100/200 μM HCA for 24 h (Figure S2B,C). Therefore, in this study, we opted to pretreat HUVECs with HCA at a concentration of 100/200 μM for 24 h prior to subjecting them to H/R conditions. Following the induction of H/R, levels of inflammatory cytokines (TNF-α, IL-1β, and IL-6) were significantly lower in the group treated with HCA compared to the untreated group (Figure S2D–F). To assess the impact of HCA on migration ability, we evaluated cell migration in both treated and untreated groups of HUVECs. Our findings revealed that pre-treatment with HCA significantly enhanced the migratory capacity of HUVECs compared to the H/R group (Figure 4A,B). Furthermore, The Transwell results also confirmed the promoting effect of HCA on the migration ability of HUVECs induced by H/R (Figure 4C,D). Increased endothelial permeability is a significant contributor to the development of pulmonary IRI, leading to the formation of an endothelial gap [3]. VE-cadherin, localized on the cell surface, and ZO-1 serve as cytoskeletal connectors that play a crucial role in modulating intercellular adhesion, which is essential for increasing cellular permeability [26].To confirm the impact of H/R on endothelial barrier damage, immunofluorescence analysis was conducted to assess the protein expression levels of VE-cadherin and ZO-1. In the control group, VE-cadherin and ZO-1 were evenly distributed along the cell membrane, exhibiting a stitch-like structure. However, compared with the control group, H/R induced visible leakage in endothelial cells, with multiple disconnected openings observed at cell junctions. Treatment with HCA increased both VE-cadherin and ZO-1 levels while improving their intercellular distribution (Figure 4E,F). Following H/R stimulation, mRNA expression of both VE-cadherin and ZO-1 significantly decreased but could be restored by pretreatment with HCA (Figure 4G).

### 3.5. Enhanced Protection against H/R-Induced Oxidative Stress in HUVECs Is Observed upon Administration of HCA

**Figure 5:**
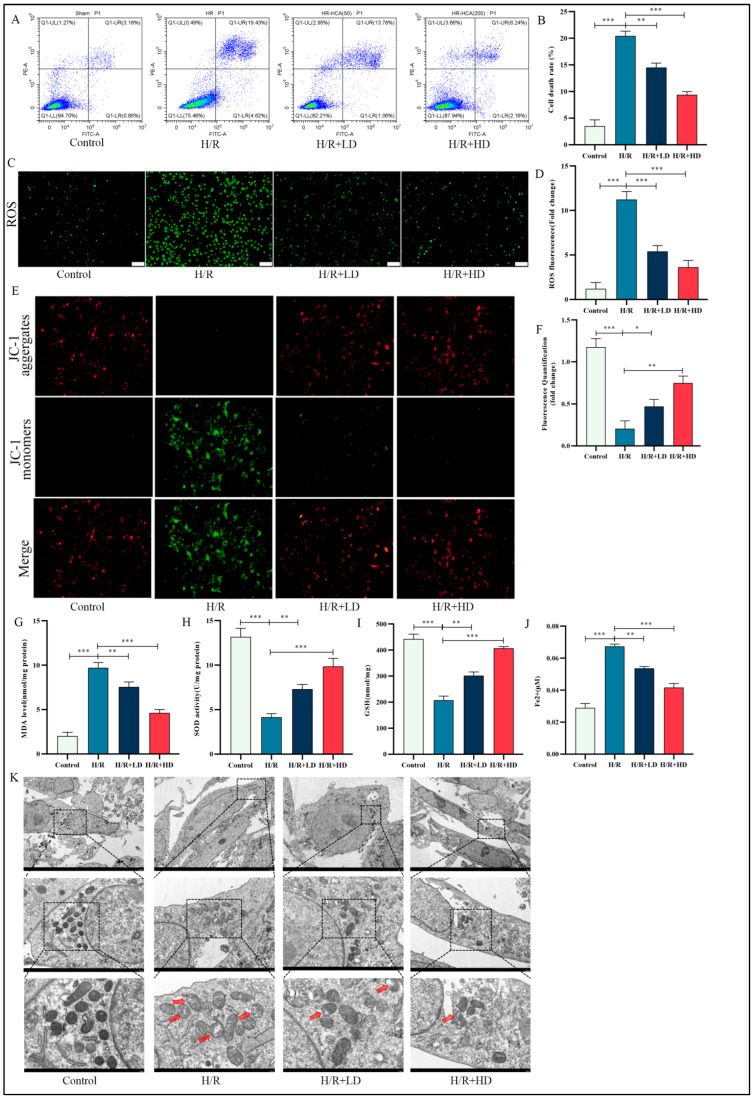
HCA supplementation markedly suppressed ROS generation, the collapse of mitochondrial membrane potential, and iron accumulation in HUVEC cells after H/R stimulation. (**A**) The cell death rate of HUVEC was detected via flow cytometry. (**C**) Representative images of fluorescence probe for ROS (bar: 100 μM). *n* = 5 per group. (**E**) Representative images of fluorescence probe for mitochondrial membrane potential determined by JC-1 (bar: 50 μM). (**B**,**D**,**F**) Quantification of the results of (**A**,**C**,**E**). (**G**–**J**) Relative MDA, SOD, GSH, and Fe^2+^ content levels in the indicated group. (**K**) HCA improved mitochondrial ultrastructure as observed by TEM (scale bar = 5 μM, 2 μM, and 1 μM). Red arrow, damaged mitochondria.* *p* < 0.05; ** *p* < 0.01; *** *p* < 0.001.

Flow cytometry analysis revealed that H/R stimulation notably increased the cell death rate in HUVECs, whereas this effect was attenuated by treatment with HCA (Figure 5A,B).The overactivation of oxidative stress leads to lipid peroxidation and plays a crucial role in the initiation and progression of ferroptosis. HCA significantly attenuated the ROS level induced by H/R, with the high-dose treatment demonstrating superior efficacy (Figure 5C,D). Abnormal changes in mitochondrial membrane potential are indicative of mitochondrial dysfunction, which is widely recognized as a hallmark of ferroptosis. To investigate HUVECs ferroptosis, we performed JC-1 staining. As depicted in Figure 5E,F, following H/R stimulation, there was a dissociation of JC-1 aggregates (reflected by decreased red fluorescence intensity) into JC-1 monomers (reflected by increased green fluorescence intensity). This resulted in a notable decline in the ratio of red/green fluorescence intensity, indicating an exacerbation of mitochondrial damage. Remarkably, administration of HCA partially restored the decrease or loss of mitochondrial membrane potential. Additionally, our results demonstrated that HCA significantly reduced MDA and Fe^2+^ levels while increasing SOD and GSH levels as influenced by H/R stimulation (Figure 5G–J). We further evaluated chondriosome morphological characteristics using TEM and observed that H/R induced significant alterations, including smaller mitochondria and reduced cristae density; however, these I/R-induced changes were ameliorated by HCA treatment (Figure 5K). Collectively, these findings suggest that H/R may induce cell ferroptosis through modulation of oxidative stress levels and mitochondrial damage in HUVEC.

### 3.6. HCA Regulated Proteins Associated with Ferroptosis in HUVECs

**Figure 6:**
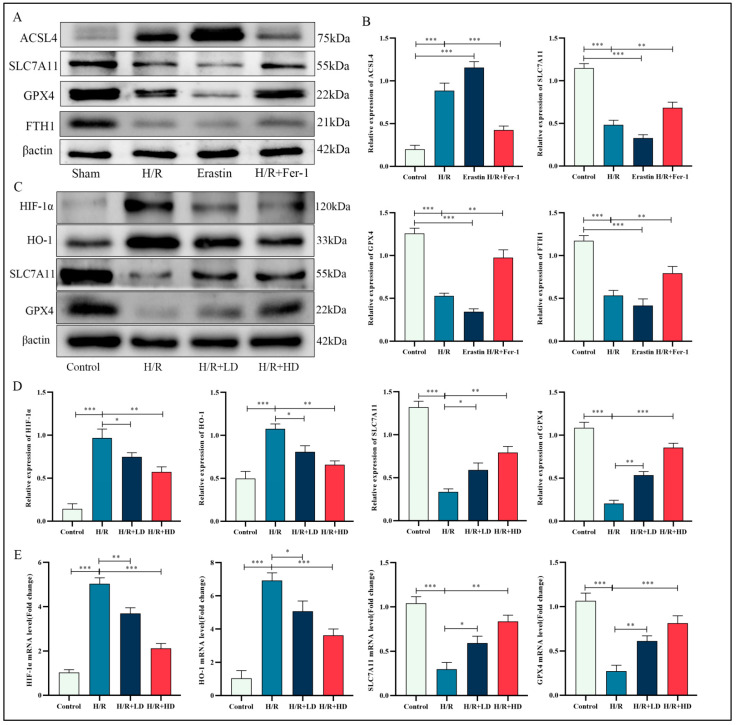
HCA supplementation markedly suppressed ferroptosis in HUVEC cells after H/R stimulation. (**A**) Immunoblots for ASCL4, SLC7A11, GPX4, and FTH1 in the indicated group treated either with erastin (4 μM) or Fer-1 (1 μM). (**C**) Western blots for HIF-1α, HO1, SLC7A11, and GPX4 protein of HUVEC cells in the indicated group. (**B**,**D**) Quantification of the results of (**A**,**C**). (**E**) Relative mRNA expression of HIF-1α, HO-1, SLC7A11, and GPX4 in the indicated group. *n* = 5 per group. (Data are presented as mean ± SD. * *p* < 0.05, ** *p* < 0.01, and *** *p* < 0.001 compared with indicated group).

The molecular mechanisms underlying the protective effects of HCA on H/R-induced lung injury were investigated using a Western blot assay. The results revealed that H/R treatment led to an increase in ACSL4 expression and a significant decrease in SLC7A11, GPX4, and FTH1 expressions, similar to erastin treatment, which is known to induce ferroptosis. However, pretreatment of HUVECs exposed to H/R with Fer-1 resulted in an apparent increase in SLC7A11, GPX4, and FTH1 expressions while decreasing ACSL4 expression (Figure 6A,B). Fer-1, a well-known ferroptosis inhibitor that blocks lipid peroxidation, was used for this purpose. These findings confirm the occurrence of ferroptosis during H/R. Furthermore, increasing doses of pretreatment with HCA on HUVECs exposed to H/R significantly upregulated SLC7A11 and GPX4 expressions. Our study also demonstrated that the expression levels of both HIF-1α and HO-1 were significantly upregulated after exposure to H/R. However, pretreatment with HCA decreased their expressions (Figure 6C,D). HCA substantially increased the mRNA levels of SLC7A11 and GPX4 while decreasing those of HIF-1α and HO-1, which were elevated or reduced, respectively, due to H/R (Figure 6E). Therefore, it can be concluded that inhibition of ferroptosis by HCA may be mediated through the HIF-1α and HO-1 pathway.

### 3.7. The Effect of HCA on H/R-Induced Ferroptosis in HUVECs Was Abolished by Overexpression of HIF-1α

**Figure 7:**
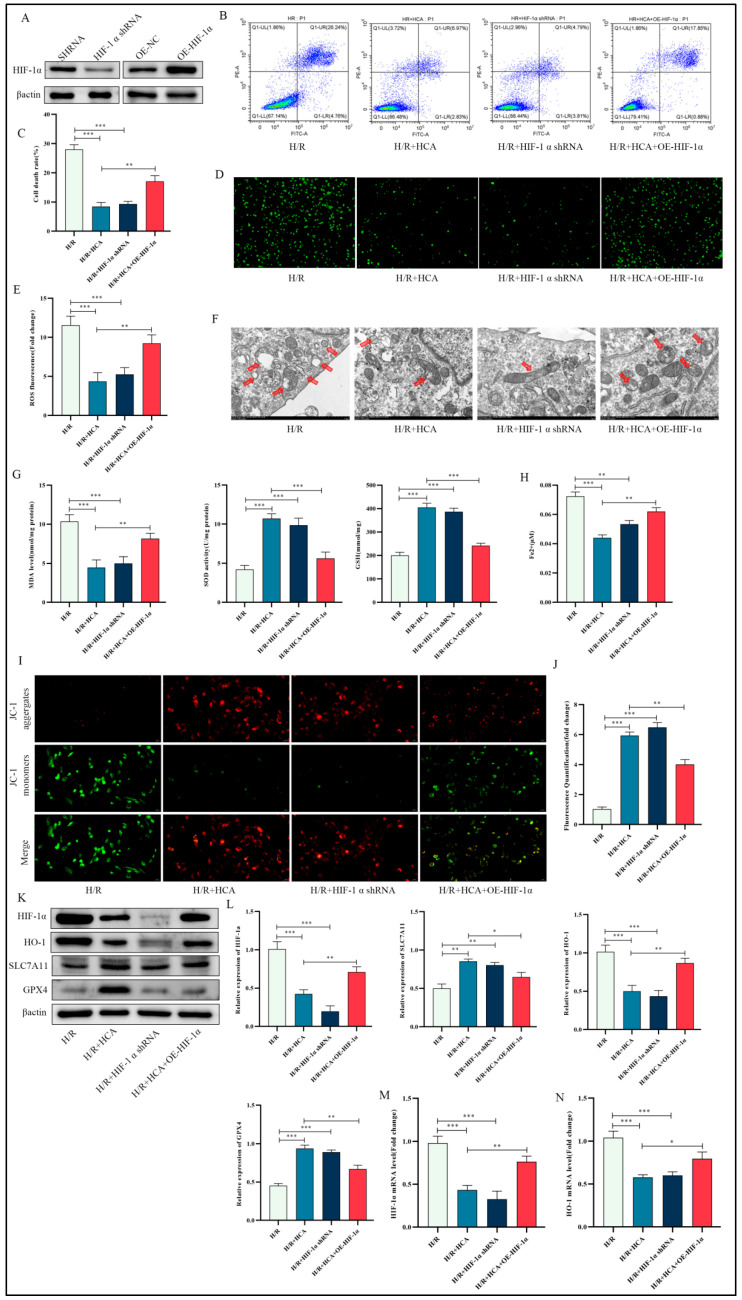
HCA inhibits H/R induced ferroptosis through HIF-1α-dependent pathways in HUVEC cells. HIF-1α protein levels were detected by Western blotting (**A**). The cell death rate of HUVEC was detected via flow cytometry (**B**,**C**). Representative images of fluorescence probe for ROS (bar: 100 μM) (**D**,**E**). (**F**) HCA improved mitochondrial ultrastructure as observed by TEM (scale bar = 1 μM). Red arrow, damaged mitochondria. Relative MDA, SOD, GSH, and Fe^2+^ levels in the indicated group (**G**,**H**). Representative images of fluorescence probe for mitochondrial membrane potential determined by JC-1 (bar: 20 μM) (**I**,**J**). Western blots for HIF-1α, HO1, SLC7A11, and GPX4 protein of HUVEC cells in the indicated group (**K**,**L**). Relative mRNA expression of HIF-1α and HO-1 in the indicated group (**M**,**N**).* *p* < 0.05; ** *p* < 0.01; *** *p* < 0.001.

Cells were infected with a lentiviral vector expressing shRNA targeting HIF-1α. HIF-1α overexpression was conducted in HUVEC cells by using cell transfection. We performed Western blotting to confirm the knockdown effect and the transfection efficiency. The results suggested that HIF-1α expression was significantly decreased and pronouncedly overexpressed individually (Figure 7A). The analysis of flow cytometry described that, similar to HCA pretreatment knockdown, HIF-1α led to significantly lower levels of cell death rate relative to the H/R group, which was partially reversed by overexpression of HIF-1α (Figure 7B,C). Consistent with previous findings, HIF-1α knockdown reduced ROS, MDA, and Fe^2+^ levels and ameliorated H/R-induced mitochondrial morphological changes, while SOD and GSH levels were increased compared to the H/R group, all of which were reversed by HIF-1α overexpression (Figure 7D–H). Detection of mitochondrial membrane potential showed that HCA combined with HIF-1α overexpression significantly decreased the ratio of red/green fluorescence intensity, indicating an increase in the degree of mitochondrial damage (Figure 7I,J). Furthermore, HIF-1α knockdown increased SLC7A11 and GPX4 expression and decreased HO-1 expression relative to the H/R group. However, after overexpression of Hif-1α, the Hif-1α and HO-1 were significantly upregulated, while the SLC7A11 and GPX4 expression was downregulated compared with H/R-HCA group (Figure 7K,L). The changes in mRNA levels of Hif-1α and HO-1 were consistent with the protein changes (Figure 7M,N). These results hint that Hif-1α inhibition is essential for the beneficial effect of HCA on LIRI.

## 4. Discussion

**Figure 8:**
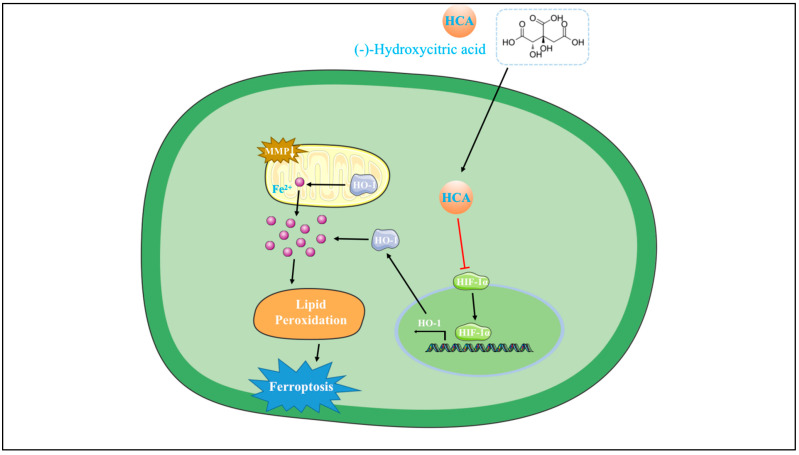
Graphical abstract of HCA alleviating LIRI.

In this study, we investigated the therapeutic impact of HCA on LIRI using a mouse model and delved into its mechanism of action on I/R-induced HUVECs through cell experiments. Our experimental results illustrated that HCA treatment effectively alleviated pulmonary injury and suppressed the inflammatory response triggered by I/R. Furthermore, HCA treatment led to a reduction in the accumulation of lipid peroxidation and Fe^2+^ in lung tissues subjected to I/R. Disturbances associated with ferroptosis during the I/R process were observed in lung tissue, but these were ameliorated by pretreatment with HCA. Additionally, HCA pretreatment significantly enhanced endothelial cell permeability, inhibited the breakdown of mitochondrial membrane potential, decreased ROS generation and iron content, and mitigated lipid accumulation in vivo. The inhibition of lung endothelial ferroptosis during LIRI by HCA was mediated through the suppression of the Hif-1α pathway (Figure 8).

The widespread application of HCA is rooted in its versatile pharmacological properties, which encompass anti-inflammatory, anti-obesity, and anti-cancer effects [13]. Through the activation of the adiponectin–AMPK signaling pathway, HCA effectively mitigated lipid droplet accumulation, enhanced glucose catabolism, and expedited energy metabolism in broiler chickens [27]. Additionally, HCA has demonstrated neuroprotective effects against nerve injury induced by multiple sclerosis by alleviating inflammation and oxidative stress [13]. In our study, we observed that pretreatment with HCA significantly mitigated pulmonary inflammation, oxidative stress, and edema in lung tissue subjected to LIRI as well as in HUVEC. Based on these findings, we propose that HCA holds promise as a potential therapeutic candidate for LIRI.

Ferroptosis, a regulated form of cell death, is characterized by the accumulation of lipid hydroperoxides to lethal levels in an iron-dependent manner [28]. Iron and its derivatives play crucial roles as active centers in numerous enzymes involved in ROS production; excessive ROS levels can result in damage to deoxyribonucleic acid (DNA), proteins, and lipids, ultimately leading to cell death [29]. I/R is a pathological condition associated with increased iron levels that have been suggested to mediate tissue damage during I/R [30]. Consistent with this notion, the inhibition of ferroptosis has been shown to reduce I/R injury in various animal models of I/R [31,32,33]. Therefore, regulators of ferroptosis represent potential therapeutic targets for I/R injury. In this study, we observed ferroptosis-like morphological changes along with elevated Fe^2+^ levels, ROS levels, and ferroptosis-associated protein expression in lung tissue and HUVEC following I/R. However, these effects were attenuated by post-treatment with HCA. These findings suggest the involvement of ferroptosis in LIRI and demonstrate that posttreatment with HCA mitigates I/R-induced ferroptotic cell death.

Hypoxia-inducible factor (HIF) plays a pivotal role in the adaptive response to hypoxia by activating genes involved in oxygen homeostasis and metabolism [34]. Intracellular oxygen levels tightly regulate HIF-1α protein stability, subcellular localization, and transcriptional activity [35]. The half-life of HIF-1α is extremely short under normal oxygen levels: less than 5 min; however, it undergoes significant upregulation during tissue ischemia. The complexity and significance of the HIF-1α pathway have been demonstrated through the identification of numerous downstream target genes regulated by HIF-1α. Although the expression of HIF-1α is crucial under hypoxic conditions, it may have detrimental effects on vascular responses, inflammatory processes, and oxidative stress levels following ischemic conditions [36]. A previous study showed that the Bu Yang Huan Wu decoction can mitigate reperfusion injury after ischemic stroke in rats by inhibiting HIF-1α and VEGF while promoting β-ENaC expression [37]. In the context of hypoxia, elevated levels of HIF-1α could induce upregulation of HO-1 as a downstream target gene. This induction could trigger ferroptosis through the release of ferrous iron [38]. Cellular heme undergoes oxidation by HO-1, producing carbon monoxide, biliverdin, and free iron, which collectively play a critical role in responding to oxidative stress. Indeed, HO-1 exhibits a dual role in IRI [38]. On one hand, HO-1 mediates ferroptosis by promoting the accumulation of free iron and exacerbating oxidative stress, thereby leading to critical ROS accumulation and triggering tissue damage. On the other hand, HO-1 also functions as an antioxidant enzyme that plays a cytoprotective role through the conversion of heme into the antioxidant biliverdin. In this study, both I/R and H/R stimulation significantly upregulated the expression of HIF-1α and HO-1 in lung tissue and HUVECs, suggesting that during I/R or H/R, HO-1 exerts a pro-ferroptotic effect. The Western blot assay confirmed the upregulation of Hif-1α, HO-1, and ACSL4 as well as the downregulation of SLC7A11, GPX4, and FTH1. However, treatment with HCA increased levels of key ferroptosis-related proteins including GPX4 and SLC7A11 while decreasing levels of Hif-1α and HO-1. Knockdown of HIF-1α significantly improved apoptosis and oxidative stress in HUVEC cells similar to what was observed with HCA treatment. Conversely, overexpression of Hif-1α counteracted the inhibitory effects on endothelial cell ferroptosis induced by H/R mediated by HCA, suggesting that inhibition of Hif-1α is crucial for protection against HCA. Furthermore, it should be noted that this study has certain limitations: prophylactic administration was used in this study; however, since LIRI is a non-selective pathophysiological process, preventing it in advance can be challenging.

## 5. Conclusions

In conclusion, both I/R and H/R can induce oxidative stress and ferroptosis in the lungs of mice. However, pretreatment with HCA may effectively attenuate lung injury and ferroptosis by inhibiting the Hif-1α pathway, as demonstrated in both in vitro and in vivo experiments. Therefore, HCA holds promise as a potential therapeutic candidate for preventing LIRI in mice.

## Data Availability

The data that support the study are available from the corresponding author upon reasonable request.

## Supplementary Materials

The following supporting information can be downloaded at: https://www.mdpi.com/article/10.3390/cimb45120616/s1, Figure S1: Therapeutic dose of HCA displayed no organic toxicity; Figure S2: HCA attenuates H/R-induced HUVEC inflammation; Table S1: The primers used in real-time PCR.
