# Supplementary material for: Hydroxycitric Acid Alleviated Lung Ischemia-Reperfusion Injury by Inhibiting Oxidative Stress and Ferroptosis through the Hif-1α Pathway
Source: Curr Issues Mol Biol. 2023 Dec 8;45(12):9868–86. doi: 10.3390/cimb45120616 (PMC10742043; doi:10.3390/cimb45120616)
Supplement: Supplementary file 1 [file cimb-45-00616-s001.zip › cimb-2728156-supplementary.pdf]

Supplementary Materials

Table S1 The primers used in real-time PCR

Species	Gene	Forward primer	Reverse primer
Mice	*TNF-α*	*ACTGAACTTCGGGGTGATCGGT*	*TGGTTTGCTACGACGTGGGCTA*
Mice	*IL-6*	*CCCCAATTTCCAATGCTCTCC*	*CGCACTAGGTTTGCCGAGTA*
Mice	*IL-1β*	*AATGAAGGAACGGAGGAGCC*	*CTCCAGCCAAGCTTCCTTGT*
Mice	*β-actin*	*ACTCCACTCACGGCAAATTC*	*TCTCCTATGGTGGTGACGACA*
Human	*TNF-α*	*TCCAGGCGGTGCTTGTTC*	*GCTTGTCACTCGGGGTTC*
Human	*IL-6*	*AGCCCTGAGAAAGGAGACA*	*CCAAAAGACCAGTGATGAT*
Human	*IL-1β*	*CGGGACTCACAGCAAAAAA*	*TTCAACACGCAGGACAGGT*
Human	*HIF-1α*	*GAACGTCGAAAAGAAAAGTCTCG*	*CCTTATCAAGATGCGAACTCACA*
Human	*HO-1*	*GCCAGCAACAAAGTGCAAGA*	*TAAGGACCCATCGGAGAAGC*
Human	*SLC7A11*	*TGTGGGGTCCTGTCACTATTTG*	*GATATCACAGCAGTAGCTGCAGG*
Human	*GPX4*	*GGCAAGACCGAAGTAAACTACAC*	*ACATATCGAATTTGACGTTGTAGC*
Human	*ZO-1*	*CAACATACAGTGACGCTTCACA*	*CACTATTGACGTTTCCCCACTC*
Human	*VE-Cadherin*	*TTGGAACCAGATGCACATTGAT*	*TCTTGCGACTCACGCTTGAC*
Human	*β-actin*	*ACAGAGCCTCGCCTTTGC*	*CCACCATCACGCCCTGG*

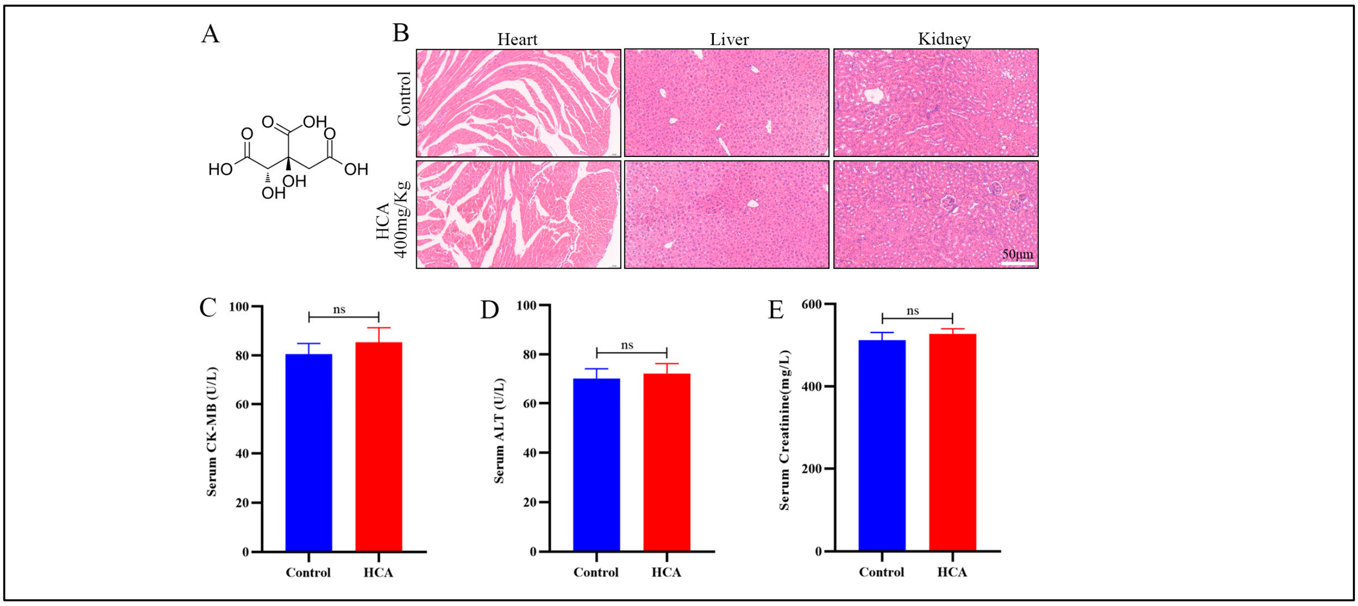

Figure S1. Therapeutic dose of HCA displayed no organic toxicity. (**A**) Molecular structure formula of (-) -Hydroxycitric acid. (**B**) Representative images of H&E staining of heart, liver and kidney tissue.Morphology was observed using light microscopy. (bar: 100 μM) (**C**) The levels of CK-MB, ALT and creatinine in serum."*" indicates significant differences between the different groups: *P < 0.05, **P < 0.01, ***P < 0.001, ns, no significance.

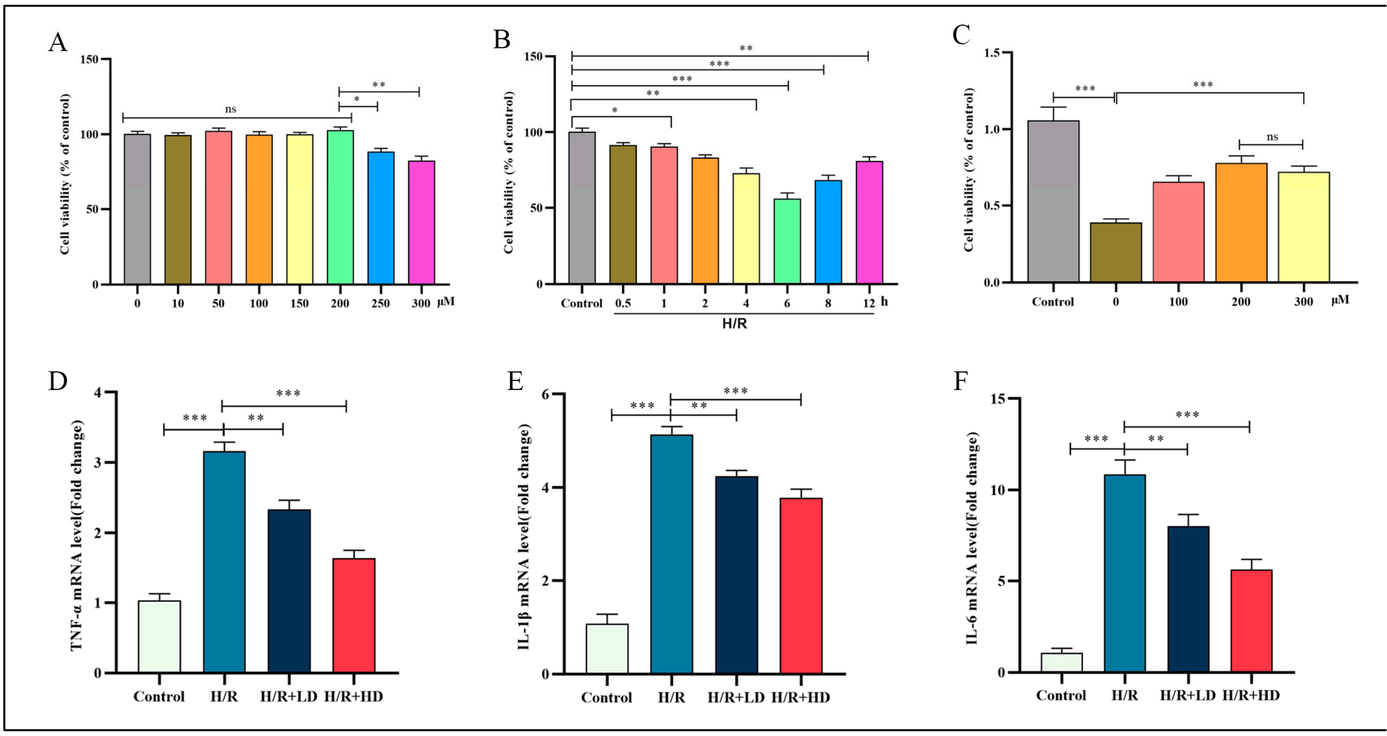

Figure S2. HCA attenuates H/R-induced HUVEC inflammation (**A**) Cell viability was measured with a CCK-8 assay after treatment of HCA at various concentrations (0, 10, 50, 100, 150, 200, 250 and 300 μM). (**B**) Cell viability was measured by CCK-8 assay at different reoxygenation times (0, 0.5, 1, 2, 4, 6, 8 and 12 h). (**C**) The cells were treated with different concentrations of HCA (100, 200, 300 μM), then the hypoxia (12h) and reoxygenation (6h) models were constructed. The cell viability was measured by CCK-8 assay. (**D**–**F**) The mRNA level of TNF-α, IL-1βand IL-6 in the indicated groups.*P < 0.05, **P < 0.01, ***P < 0.001, ns, no significance.
